# Validity of ultrasonography-derived predictions for estimating skeletal muscle volume: a systematic literature review

**DOI:** 10.1186/s12880-021-00638-9

**Published:** 2021-07-05

**Authors:** Rasmus Liegnell, Fredrik Wessman, Adel Shalabi, Marita Harringe

**Affiliations:** 1 Stockholm Sports Trauma Research Centre, Department of Molecular Medicine and Surgery, Karolinska Institute, Stockholm, Sweden; 2 Centre for Medical Imaging, University Hospital, Uppsala University, Uppsala, Sweden

**Keywords:** Bland–Altman analysis, Magnetic resonance imaging, Muscle thickness, Muscle volume, Prediction equation, Ultrasonography

## Abstract

**Background:**

The amount of muscle volume (MV) varies between individuals and is important for health, well-being and performance. Therefore, the monitoring of MV using different imaging modalities is important. Magnetic resonance imaging (MRI) is considered the gold standard, but is not always easily accessible, and the examinations are expensive. Ultrasonography (US) is a much less expensive imaging method widely used to measure changes in muscle thickness (MT). Whether MT may translate into MV needs further investigation.

**Purpose:**

The aim of this review is to clarify whether US-derived equations based on MT predict MV based on MRI.

**Methods:**

A systematic literature review was conducted according to the PRISMA statement, searching the electronic databases PubMed, CINAHL and Web of Science, for currently published equations to estimate MV with US.

**Results:**

The literature search resulted in 363 citations. Twelve articles met the eligibility criteria. Ten articles scored eight out of eleven on QUADAS and two scored nine. Thirty-six prediction equations were identified. R values ranged between 0.53 and 0.961 and the standard error of the estimate (SEE) ranged between 6 and 12% for healthy adult populations, and up to 25.6% for children with cerebral palsy. Eight studies evaluated the results with a Bland–Altman plot and found no systematic errors. The overall strength and quality of the evidence was rated “low quality” as defined by the GRADE system.

**Conclusions:**

The validity of US-derived equations based on MT is specific to the populations from which it is developed. The agreement with MV based on MRI is moderate with the SEE ranging between 6 and 12% in healthy adult populations. Suggestions for future research include investigations as to whether testing positions or increasing the number of measuring sites could improve the validity for prediction equations.

## Background

Skeletal muscle accounts for about 40% of total body weight. Its primary function is to generate force and create physical movement essential for everyday living, health and performance [1]. Skeletal muscle is also an endocrine organ, secreting a collection of factors called myokines that seem to have positive health effects on a variety of organs throughout the body [2]. The amount of muscle mass or muscle volume (MV) varies between individuals and is influenced by a complex interaction between nutrition, physical load, hormones, age, injuries and diseases [1]. MV gradually declines with age, which eventually may lead to sarcopenia, affecting 10% to 50% of individuals above 65 years of age [3]. Sarcopenia is associated with an increased risk of being hospitalized and all-cause mortality [4, 5]. On the other hand, an increased or high amount of total MV seems to be protective and reduce the likelihood of common diseases and disabilities like cardiovascular disease, diabetes and immobility [6–8].

MV is strongly correlated with the ability to produce force and is therefore a good predictor for strength, through the ability to create joint torque (force x moment arm) [9, 10]. Decreased MV and reduced strength are common after an injury, surgery, or immobilization. Meier et al. [11] reported that after knee arthroplasty an inability to activate quadriceps contributed to the loss in strength the first few months and that quadriceps MV was a strong predictor of strength after more than one year. Quadriceps MV is also predictive of patient-reported function and persistent strength deficit after anterior cruciate ligament (ACL) reconstruction [12].

Hypertrophy is often seen as a way to improve strength in performance, rehabilitation or the activities of daily living. One of the main outcomes after repeated sessions of loading, through exercise or heavy daily activities, is the growth of contractile proteins within the skeletal muscle, leading to hypertrophy and an increase in MV [13]. The skeletal muscle is a plastic tissue that constantly adapts to the exposure and requirements in life. Therefore, valid measurements of MV and the changes in mass over time are of great interest in order to ensure that the intervention causes hypertrophy and muscle growth. Direct measurement of the changes in protein synthesis is possible but requires muscle biopsies and expensive tracers [14, 15]. Measurement of MV is achievable with high validity via the water displacement method [16] but this requires that the muscle be removed from its owner making it impossible to measure living beings and changes between different occasions.

Imaging is a useful tool to reduce suffering and enable non-invasive measurement of MV. Magnetic resonance imaging (MRI) or computed tomography (CT) are considered the gold standard [17]. MRI is preferable since CT involves radiation. The method for estimating MV measured with MRI (MV_MRI_) is determined by measuring a muscle’s single axial anatomical cross-sectional area (ACSA), in multiple sections along the entire length of the muscle, and then multiplying ACSA by the length of each section [18]. MRI is not always easily accessible, and the examinations are expensive. Therefore ultrasonography (US) has become a widely used method to measure changes in muscle thickness (MT). Several studies have measured the acute and long-term differences in MT with US, before and after a period of exercise [19–21]. MT dimensions are measured as the distance from the subcutaneous adipose tissue muscle interface to the muscle bone interface [22]. MT is well correlated to the MRI cross-sectional area (CSA) in both the lower [23] and upper extremities [24].

Estimating MV with US (MV_US_) is commonly based on MT measurement and is achieved by developing prediction equations through multiple regression analysis including limb length or other anthropometric variables [9, 25]. The true value of MV is unknown but since MRI is considered the gold standard, it would be best if the results from MV_US_ and the results from MV_MRI_ were the same. When comparing MV_US_ to the water displacement method, standard error of the estimate (SEE) between 10 and 13% have been reported [16]. Similar SEE percentages are reported when MV_MRI_ and MV_US_ are compared [25]. Even though SEE varies, the correlation in a population should be good, since both methods aim to measure the same thing [26]. If the more accessible US can estimate MV in a satisfying manner it would be valuable to the clinician.

Therefore, the aim of this study was to perform a systematic literature review with the purpose of collecting the currently published equations to calculate MV_US_ and clarify how well US-derived equations based on muscle thickness predict MV_MRI_.

## Methods

### Search strategy

The study was conducted according to the PRISMA statement [27]. A systematic search took place on the 30th of January, 2020, in the electronic databases PubMed, CINAHL and Web of Science. MeSH terms were identified and used whenever possible. MeSH terms “ultrasonography” and “magnetic resonance imaging” were used as a concept and combined with Boolean operator AND. Search terms “muscle thickness” and “muscle volume” were used as a concept and combined with Boolean operator OR. Both concepts were combined with Boolean operator AND. Investigators (RL and FW) screened the titles of all articles identified and, if eligible, the abstracts were read and discussed. Unless both investigators agreed that the study did not meet the eligibility criteria, the study was included for full text review. There was consensus between both investigators regarding eligibility during the full text review. Reference lists of the studies included were screened for eligible literature.

### Eligibility criteria

To be included, the studies needed to meet the following criteria: 1: Measure MT with B-mode US. 2: Use US-derived equations based on MT to predict MV. 3: Use MRI as the reference method for MV. 4: Be published in the English language. Criteria for exclusion were the following: 1: Published before the year 2000. 2: Animal studies. 3: Cadaver studies. 4: Reviews.

### Quality assessment

To assess the quality of the included studies, a translated version of the Quality Assessment of Diagnostic Accuracy Studies (QUADAS) [28] published by the Swedish Agency for Health Technology, Assessment and Assessment of Social Services was used*.* Investigators (RL and FW) assessed each study independently, and thereafter discussed each study until consensus was reached. Group Reading Assessment and Diagnostic Evaluation (GRADE) was used to assess the overall strength and quality of the evidence [29].

### Ethical considerations

All the studies included declared that written or informed consent had been given by study participants. In three studies, the participants were children or adolescents below the age of 18: these studies had obtained consent from their parents [30–32]. Most included studies declared that they had received approval from an independent ethics committee, with the exception of two studies [33, 34] where there were no such declarations.

### Statistical analysis

Two Bland–Altman plots were created from the mean values identified in the included studies, with the purpose of examining the agreement between the two methods in a descriptive manner. The values reported in cm^3^ and kg were separated into different plots. Both plots were plotted against the mean value of MV_MRI_ and MV_US_ for every segment. The BIAS, standard deviation (SD), and upper and lower limits of agreement were calculated and reported as a percentage, according to the method described by Bland and Altman [26].

## Results

**Fig. 1:**
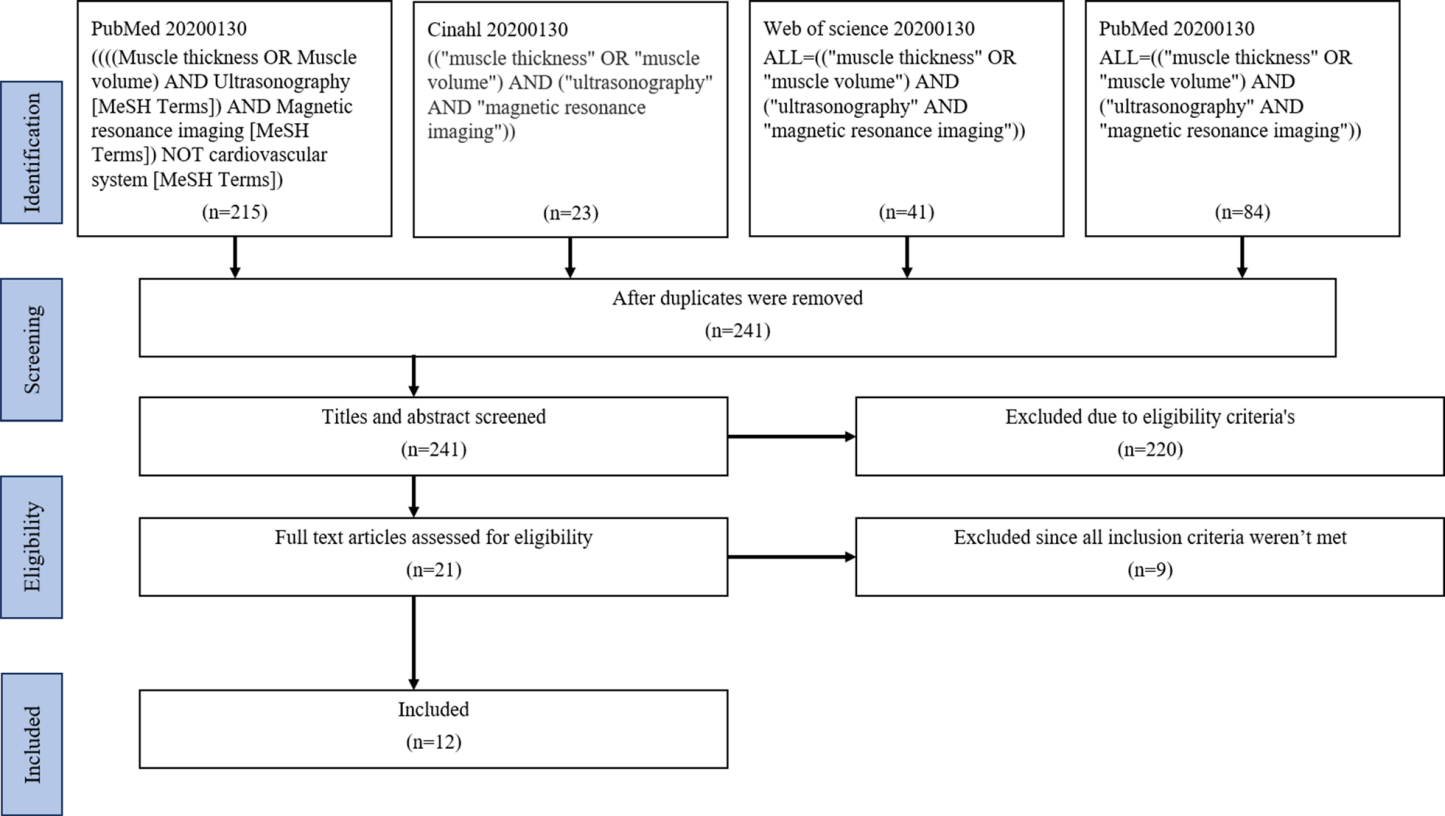
Flow chart of the literature search, based on work from The PRISMA Group [27]

The literature search resulted in 299 citations in the PubMed database, 23 in CINAHL and 41 in Web of Science. After abstracts had been analysed and discussed, 21 articles were selected for full text review. In the end, 12 articles met the eligibility criteria and were included in the systematic literature review (Fig. 1). Ten articles scored eight out of eleven on the QUADAS score and two scored nine out of eleven (Table 1). All articles lacked the same items on the QUADAS score, and stated that it was unclear whether those who analysed the index test were blinded to the results of the reference test, and vice-versa.

**Table 1:** Descriptive data

Subjects	Body part	Mean MV_MRI_ [± SEM] (± SD)	Mean MV_US_ [± SEM] (± SD)	Position MRI	Position UL	QUADAS	References
26 ♂ (23–34y)	Anterior upper arm	297.5 [14.9] cm^3^	273.6 [15.4] cm^3^	Supine	Standing	8/11	[35]
	Posterior upper arm	405.4 [22.6] cm^3^	387.9 [30.9] cm^3^				
26 ♂ (X̄ 25.7y)	Anterior upper arm			Supine	Standing	8/11	[9]
	Posterior upper arm						
46 ♂ (20–70y)	Anterior upper thigh	1637.5 (383.51) cm^3^	1660.7 (386.2) cm^3^	Supine	Standing	8/11	[33]
27 ♂ (X̄ 25.3y)	Anterior upper arm	311.5 (68.5) cm^3^		Supine	Standing	8/11	[34]
	Posterior upper arm	427.0 (112.2) cm^3^					
	Anterior upper thigh	1825.3 (428) cm^3^					
	Posterior lower leg	1072.7 (226.3) cm^3^					
72 ♂♀ (18–61y)	Total body	20.2 (6.5) kg	19.6 (6.5) kg	Supine	Standing	8/11	[37]
	Arm	2.0 (0.7) kg	1.9 (0.7) kg				
	Trunk	8.3 (2.9) kg	8.2 (2.8) kg				
	Thigh	7.7 (2.6) kg	7.5 (2.2) kg				
	Lower leg	2.2 (0.6) kg	2.2 (0.6) kg				
10 Prepubertal children	Total body	9.9 (1.4) kg	9.5 (1.1) kg	Supine	Standing	8/11	[30]
(X̄ 9.2y) ♂	Arm	0.8 (0.1) kg	0.7 (0.1) kg				
(X̄ 10.3y) ♀	Trunk	4.3 (0.7) kg	4.7 (0.6) kg				
	Thigh	3.6 (0.6) kg	3.3 (0.6) kg				
	Lower leg	1.1 (0.2) kg	0.7 (0.1) kg				
21 Adolescents	Total body	17.4 (3.8) kg	17.5 (4.3) kg				
(X̄ 14.1y) ♂	Arm	1.5 (0.4) kg	1.5 (0.4) kg				
(X̄ 13.8y) ♀	Trunk	7.3 (1.6) kg	7.5 (1.7) kg				
	Thigh	6.6 (1.5) kg	6.5 (1.8) kg				
	Lower leg	2.0 (0.4) kg	1.9 (0.5) kg				
147 ♂♀ (19–77y)	Anterior upper arm	182.2 (65.4) cm^3^	179.4 (62) cm^3^	Supine	Standing	8/11	[25]
20 ♂♀ (20–41y)	Inner upper thigh	19.70 (9.29) cm^3^		Supine	Standing	9/11	[38]
9 children* (2–6y)	Posterior lower leg medial & lateral	11.92 (9.12) cm^3^		Did not report	Prone	8/11	[32]
60 ♂ (6–12y)	Total body	9113 (2241) cm^3^	8942 (2841) cm^3^	Supine	Standing	8/11	[31]
	Arm	851 (198) cm^3^	825 (194) cm^3^				
	Trunk	3495 (795) cm^3^	3453 (780) cm^3^				
	Thigh	3579 (1026) cm^3^	3484 (986) cm^3^				
	Lower leg	1164 (295) cm^3^	1180 (323) cm^3^				
37 ♀ (6–12y)	Total body	7688 (2339) cm^3^	7804 (2461) cm^3^				
	Arm	743 (208) cm^3^	719 (232) cm^3^				
	Trunk	2798 (519) cm^3^	2982 (929) cm^3^				
	Thigh	2905 (905) cm^3^	3030 (1015) cm^3^				
	Lower leg	1084 (344) cm^3^	1074 (346) cm^3^				
60 ♂♀ (51–77y)	Anterior upper thigh	1000 (373.3) cm^3^	1019.5 (370.9) cm^3^	Supine	Standing	8/11	[39]
61 ♂ (X̄ 20.4y)	Total body	38.5 (5.8) kg	38.5 (5.7) kg	Prone	Standing	9/11	[36]

SEM, standard error of the mean. SD, standard deviation. X̄, mean in sample. y, years. ♂, boys and males. ♀, girls and females

*Nine children with bilateral involvement spastic CP (6 ♂ and 3 ♀) in total, 18 lower limbs were evaluated

In total, the studies included 591 subjects. Five studies included only men [9, 33–36]. Four studies included both men and women [25, 37–39]. Two studies included prepubertal children [30, 31]. One study also included adolescents [30] and one study included children with cerebral palsy [32]. Descriptive data are presented in Table 1.

A total of 12 different body parts or muscle groups were measured, and 36 different prediction equations were identified. Correlations between MV_US_ and MV_MRI_ were good; r values ranged between 0.53 and 0.961, and the SEE ranged between 6 and 12% for healthy adult populations and up to 25.6% for children with cerebral palsy. Regression equations and measured segments are presented in Table 2. Eight studies did further analysis with a Bland–Altman plot [25, 30, 31, 33, 34, 36, 37, 39] and they found no systematic errors.

**Table 2:** Muscle thickness sites, equations, and correlations

Segments	Equations	r	SEE cm^3^	References
EF and EE	MV_US_ = L x (π x MT/2)^2^	0.962	50.7 (7.2%)	[35]
EF	MV_US_ = 2.586 BH – 1.259 BW + 7.057 CIR + 0.524 (L x (MT)^2^) − 447.46	0.943	6–8%	[9]
EE	MV_US_ = 3.478 BH – 0.180 BW + 6.674 CIR + 0.382 (L x (MT)^2^) − 559.36	0.932		
KE	MV_US_ = (MT × 311.732) + (L × 53.346) – 2058.529	0.824*	175.6 (10.6%)	[33]
EF	MV_US_ = (MT × 117.9) + (L × 12.6) − 494	0.884*	22.1 (7.3%)	[34]
EE	MV_US_ = (MT × 98.1) + (L × 31.9) − 984.4	0.842*	40.3 (9.8%)	
KE	MV_US_ = (MT × 320.6) + (L × 110.9) − 4437.9	0.787*	198.5 (11.1%)	
APF30	MV_US_ = (MT × 219.9) + (L × 31.3) − 1758	0.832*	78.9 (7.6%)	
♂ Total body (sum of 9 MT)	MV_TOT_ = 0.641 × MT_9_ x BH − 12.087	0.96	2.24 kg	[37]
♂ Total body (sum of 6 MT)	MV_TOT_ = 0.809 × MT_6_ x BH − 4.834	0.96	1.8 kg	
♂ Arm (EF + EE + LAF)	MV_TOT_ = 0.204 × MT_arm_ x BH − 0.517	0.95	0.22 kg	
♂ Trunk (AB + SUS)	MV_TOT_ = 1.303 × MT_trunk_ x BH + 1.766	0.88	1.11 kg	
♂ Thigh (KE + KF)	MV_TOT_ = 0.639 × MT_thigh_ x BH − 2.972	0.83	1.76 kg	
♂ Lower leg (APF30 + ADF)	MV_TOT_ = 0.233 × MT_lower leg_ x BH − 1.347	0.83	0.55 kg	
♀ Total body (sum of 9 MT)	MV_TOT_ = 0.594 × MT_9_ x BH − 11.32	0.91	2.75 kg	
♀ Total body (sum of 6 MT)	MV_TOT_ = 0.831 × MT_6_ x BH − 7.992	0.88	2.88 kg	
♀ Arm (EF + EE + LAF)	MV_TOT_ = 0.132 × MT_arm_ x BH + 0.093	0.53	0.47 kg	
♀ Trunk (AB + SUS)	MV_TOT_ = 0.937 × MT_trunk_ x BH + 1.794	0.61	1.27 kg	
♀ Thigh (KE + KF)	MV_TOT_ = 0.532 × MT_thigh_ x BH − 2.638	0.81	1.39 kg	
♀ Lower leg (APF30 + ADF)	MV_TOT_ = 0.237 × MT_lower leg_ x BH − 1.534	0.77	0.61 kg	
EF	MV_US_ = 60.8 × MT + 6.48 × L − 0.709 × AGE + 51.4 × SEX − 187.4	0.909*	19.9 (10.9%)	[25]
ADD	MV_US_ = 5.51 × MT × L – 434.9	0.922		[38]
APF25 medial	MV_US_ = 2.271 × L + 15.982 × MT-41.493	0.831*	4.1 (20.6%)	[32]
APF25 lateral	MV_US_ = 1.479 × L + 13.347 × MT-28.676	0.779*	3.1 (25.6%)	
♂ Total body (sum of 9 MT)	MV_US_ = 384.96 x (MT_9_ x BH) − 3662.1	0.93*	659	[31]
♂ Arm (EF + EE + LAF)	MV_US_ = 127.09 x (MT_arm_ x BH) – 76.44	0.71*	124	
♂ Trunk (AB + SUS)	MV_US_ = 992.53 x (MT_trunk_ x BH) + 363.69	0.65*	565	
♂ Thigh (KE + KF)	MV_US_ = 463.47 x (MT_thigh_ x BH) – 1624.3	0.84*	419	
♂ Lower leg (APF30 + ADF)	MV_US_ = 176.1 x (MT_lower leg_ x BH) − 539.29	0.92*	91	
♀ Total body (sum of 9 MT)	MV_US_ = 364.87 x (MT_9_ x BH) − 3523	0.89*	731	
♀ Arm (EF + EE + LAF)	MV_US_ = 132.68 x (MT_arm_ x BH) – 139.4	0.8*	89	
♀ Trunk (AB + SUS)	MV_US_ = 658.79 x (MT_trunk_ x BH) + 953.72	0.57*	561	
♀ Thigh (KE + KF)	MV_US_ = 425.40 x (MT_thigh_ x BH) – 1506.7	0.9*	286	
♀ Lower leg (APF30 + ADF)	MV_US_ = 166.19 x (MT_lower leg_ x BH) − 439.17	0.88*	103	
KE	MV_US_ = (SEX × 267.7) + (MT × 249.3) + (L × 41.1) − 1663.7	0.91*	124.4 (12%)	[39]
Total body (sum of 9 MT)	MV_TOT_ = 0.645 x (MT_9_ x BH) − 7.821	0.96		[36]

SEE, standard error of the estimate. MV_US_, estimated muscle volume via US in cm^3^. MV_TOT_, estimated muscle volume via US in kg. MT, muscle thickness in cm obtained via US. L, length of the limb in cm. CIR, circumference of upper arm at the same site for MT measuring in cm. BH*,* body height in meters. BW, body weight in kg. SEX, refers to biological differences between males 1 and females 0. AGE, values in years

♂, boys and males. ♀, girls and females. EF, elbow flexors MT obtained at 60% of anterior arm. EE, elbow extensors, 60% posterior arm. KE, knee extensors, midpoint of anterior thigh. KF, knee flexors, midpoint of posterior thigh. APF30, ankle plantar flexors, 30% of the posterior lower leg. APF25, ankle plantar flexors, 25% of the posterior lower leg. ADF, ankle dorsal flexors, 30% of the anterior lower leg. LAF, obtained at 30% of the lateral anterior forearm. AB, abdominal obtained at a distance 2–3 cm to the right of the umbilicus. SUS, subscapular, 5 cm directly below the inferior angle of the scapula. ADD, adductor, 30% of the medial anterior aspect of the thigh

*r in square instead of r

**Fig. 2:**
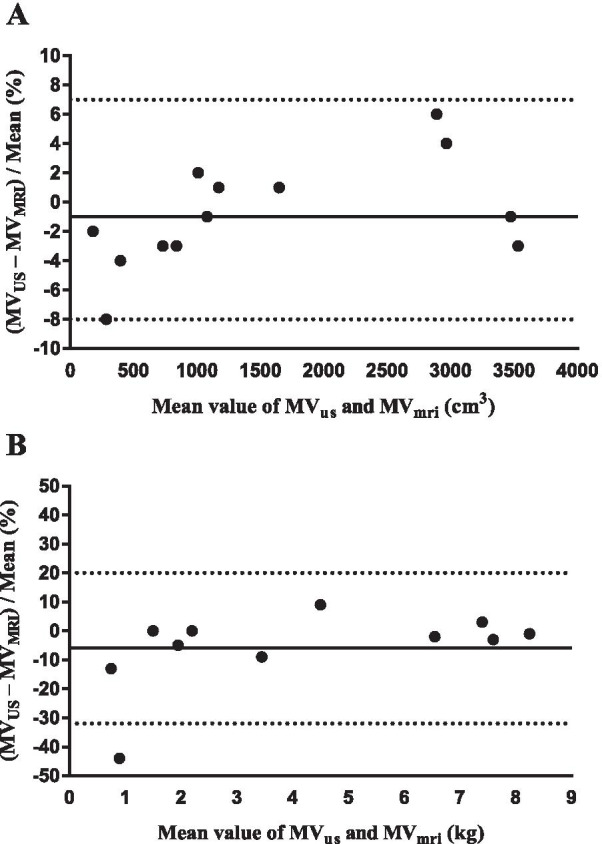
Bland–Altman agreement, differences plotted in percentages for the studies that reported MV_US_. Total body estimates are excluded in the plots. Values in plot **A** for the studies that reported data in cm^3^: Bias − 1%, SD 4%, limits of agreement upper 7% and lower − 8%. Values in plot **B** for the studies that reported data in kg: Bias − 6%, SD 13%, limits of agreement upper 20% and lower − 32%

A total of 13 segments, reported in cm^3^, from five studies [25, 31, 33, 35, 39] were included in the first plot and plotted against the average (Fig. 2A). They showed an even spread in percentage when differences between methods were plotted against the average mean. One measure crossed the lower limit of agreement, namely the anterior upper arm data reported from Miyatani et al. [35]. Three studies reported values in kg [30, 36, 37] although when total body estimates were excluded from the Bland–Altman analysis, two studies remained [30, 37]. Midorikawa et al. [30] tested the equation derived from Sanada et al. [37] and eleven segments were plotted against the average (Fig. 2B). In this plot, the data show a larger spread, illustrated by the Y-axis in the plots in Fig. 2. Two data points are the main reason for this: the arm (−44%) and lower leg (−13%) segments calculated from measures on prepubertal children reported by Midorikawa et al. [30]. The arm segment in prepubertal children crossed the lower limit of agreement.

The overall strength and quality of the evidence was rated as “low quality” as defined by the GRADE system. Two reasons for this were that eleven of twelve articles originated from the same study group and descriptive data were missing in several reports, such as means for MV [9, 32, 34, 38], and there were no individual data published in any of the studies.

## Discussion

The most important finding of this investigation was that the validity of US-derived equations based on MT is specific to the populations from which it is developed. Midorikawa et al. [30] tested the validity of MV_US_ for adolescents and prepubertal children based on equations previously derived from adults. They found inferior validity for prepubertal children, though no significant difference for adolescents. Their Bland–Altman analysis showed a relatively high level of variability for both adolescents and prepubertal children. Nakatani et al. [39] found that prediction equations developed for young adults were not valid for middle-aged and older men and women, and Toda et al. [36] showed that prediction equations derived from a sedentary population were not applicable for young male athletes. In our study, MT correlates well with MV (Table 2), neither did we find any systematic errors between the methods used to estimate MV_US_ and MV_MRI_. The SEE varied between 6 and 12% in a healthy adult population and up to 25.6% for children with cerebral palsy (Table 2).

Correlations in our review agreed with previous reviews by Abe et al. [40, 41] investigating the association between MT and MV for the upper extremity [40] and the lower extremity [41] respectively. However, Abe et al. also included studies with reference methods such as CT and cadavers [25, 33, 34, 38]. Nijholt et al. [42] conducted a systematic review investigating the validity of US-derived prediction equations to estimate MV in an elderly population aged > 60, using dual-energy x-ray absorptiometry (DEXA) as a reference. They reported r^2^ values of 0.92 and 0.96. To our knowledge, no other systematic reviews have investigated the validity of MV_US_ with MV_MRI_ as a reference.

Thirty-six different prediction equations were identified in this systematic review and the studies included in our review used different variables in their regression analysis in addition to MT (Table 2). Miyatani et al. [35] performed the first prediction equations with the formula for calculating a cylinder with limb length as a variable. The same group later reported that the prediction improved when MT was combined with limb length, compared to MT alone [34]. Eight of the studies we included used limb length as a variable in their regressions [9, 25, 32–35, 38, 39]. Limb length measurements were made with a measuring tape between anatomical landmarks and therefore represent an approximation of the actual muscle length (ML). When MV_MRI_ is determined, ML is defined as the distance between the most proximal and the most distal images in which the muscle is visible [34]. With a linear US transducer, which was used in all the included studies, a similar approach as for MV_MRI_ of measuring actual ML with repeated measurements along the limb would have been challenging and more time consuming than to determine limb length with a measuring tape. However, it is possible for an experienced sonographer to use more precise landmarks for measuring ML, by identifying the origin and insertion of specific muscles. This is still more time consuming but interesting if the length and thickness of specific muscles were to be compared instead of the limb length and thickness of a muscle group. Body height (BH) was used to express the length factor of the muscle in three of the equations [31, 36, 37], and one study, Fukunaga et al. [9], used both BH and limb length as variables.

Miyatani et al. [34] reported that the relative contribution of limb length to predict the measured MV in the multiple regression equations varied from 18% for the elbow flexors to 37.7% for the knee extensors, which was less than the MT contribution. Akagi et al. [25] included both sex and a wider range of ages when reporting the relative contribution for the elbow flexors. They found that the contribution of MT predicting MV was about 2.5 times higher than the contribution of limb length (13.6%). Also, the relative contribution of sex to predict MV (34.3%) was nearly equal to that of MT (33.9%) and that a decrease in MV did not correspond to a decrease in MT with ageing when the sex variable was statistically controlled for. Park et al. [32] noticed that the relative contribution of limb length for ankle plantar flexors’ medial and lateral head was 62.9% and 59.1% in the MV_US_ prediction based on MT in two- to six-year-old children suffering bilateral spasticity. They also conducted a multiple regression model for predicting MV based on ACSA and reported that the relative contribution of limb length for predicting MV was 24.8% for the medial head and 18.0% for the lateral, while ACSA contributed with 65.6% and 67.8%, respectively. It is not surprising that MT contributes the least in the group of young children with impaired muscle function. Children with unilateral spastic cerebral palsy have, on average, smaller volume on their affected side compared to the less affected side [43]. This, in combination with smaller mean fibre size and smaller CSA in children’s muscle mass due to the larger proportion of Type 1 muscle fibres [44], probably explains why the contribution of limb length was superior to MT in the study by Park et al. [32].

Despite the different variables included in the regression equations, there is no clear difference in SEE values, with the exception of the study on children with cerebral palsy reporting SEE of 20.6% for the medial gastrocnemius and 25.6% of the lateral gastrocnemius [32]. The children suffered bilateral spasticity, making it hard to standardise the joint positions for the measurements. It is important to standardise joint position because it will influence the muscle’s architecture [45]. The standardisation procedure used by Park et al. [32] was in the prone position with the ankle in resting position. Resting position may vary within and between subjects depending on the severity of the spasticity and Park et al. [32] suggested that better standardisation of the MT measurement in children with cerebral palsy is required. Miyatani et al. [34] measured the plantar flexors in a standing position in healthy adults, thereby making sure that the ankle joint was in the same position for every measurement, leading to a SEE of 7.6%. Considering only healthy adults would leave us with the range of SEE 6–12% and thus, less variation across the studies we included.

Developing accurate prediction equations based on MT is complex. One factor that may contribute to this complexity is that the measurement of MT with US does not differentiate between contractile and non-contractile intramuscular tissue (NCIT), while the method for MV_MRI_ excludes NCIT when digitizing the images [34]. NCIT refers to intramuscular adipose tissue and intramuscular connective tissue, and is influenced by different factors including comorbidities, age, and physical activity [46, 47]. Increased age is associated with an increase in the relative amount of NCIT within the muscle [48]. Comorbidities and inactivity are associated with increased NCIT, whereas exercise is associated with reduced levels of NCIT [46, 47].

Moreover, the changes in MV do not only depend on MT, but also on muscle width [25] and fascicle length [49]. This is especially relevant in the context of differences in pinnation angle of individual muscle, leading to a discrepancy between ACSA and physiological CSA (PCSA) [50]. ACSA represents the CSA of the muscle perpendicular to its longitudinal axis and does not represent the CSA perpendicular to all fibres in a pinnate muscle. PCSA refers to the CSA perpendicular to the fascicle plane and represents the total CSA of all the muscle fibres within the muscle [51]. PCSA is proportional to muscle force [52], increases with a larger angle of pinnation and is usually calculated from the ratio of MV to fascicle length, multiplied by the cosine of pinnation angle [50]. Aagaard et al. [53] reported that after 14 weeks of resistance exercise, vastus lateralis fibre pinnation angle increased in eleven untrained males. This allowed PCSA of single muscle fibres and thereby maximal force generating capacity to increase significantly more (+ 16%) than ACSA and MV (+ 10%). Consequently, changes in PCSA caused by exercise or inactivity may not automatically reflect the change in ACSA and MV [53]. Narici et al. [54] described that ageing is associated with reduced fascicle length and pinnation angle which could result in a decrease of PCSA, an alternation expected to have implications for muscle function [54]. Both the length of the muscle fascicles and the pinnation angle can be measured using US. One limitation is the relatively small field of view, making it hard to measure the fascicle length in certain muscles without some degree of estimation [55]. None of the equations in our study included pinnation angles or fascicle length but taken together with NCIT, this may, to some extent, explain why our review indicates that US-derived prediction equations are specific to the population from which they are derived. In order to develop a more generalised prediction equation, we believe it is important to account for comorbidities, age, sex and physical activity levels.

The agreement between two methods is illustrated in our Bland–Altman plot (Fig. 2). To minimize the influence of the variation in size of segments, the differences were plotted in percentages [56]. Furthermore, the total body data were excluded due to the large values that would have displaced values on the X-axis, and thereby been unrepresentative for the segment data. Whether to plot against the average or against the reference is debatable [57, 58]. If MRI is considered the gold standard, and the purpose is to develop another method to reach agreement with MRI, plotting against the reference seems to be more appropriate. On the other hand, with an unknown true value for MV, plotting against the average mean is most likely accurate. Bland and Altman suggest that plotting differences against the standard method might be misleading and to plot against the average is more correct in almost all applications for medical measurements [57]. Since the manual slice-by-slice segmentation technique to measure MV_MRI_ has, to our knowledge, only been validated against the water displacement method in one study [59], the choice of plotting against the average is preferable. Figure 2B illustrates data from only two studies, and Midorikawa et al. [30] tested the equation derived from Sanada et al. [37] on different populations. Consequently, the strength of Fig. 2B is that the same equation was used. However, the downside is that the equation was not derived for prepubertal children and adolescents, resulting in a larger BIAS (−6%) compared with the data in Fig. 2A (− 1%). Figure 2A is the exact opposite to Fig. 2B where different equations are mixed, but they are derived for a specific population, resulting in a better outcome.

When conducting an MRI scan, the subject is commonly placed in a supine position, even though it is possible to scan subjects in an upright position [60]. In the present review, almost all studies placed their subjects in a supine position when measuring MV_MRI_ (Table 1) but placed their subjects in a standing position when measuring MT (Table 1). We do not know the reason for this. It is also unclear whether this has any significance for the validity of MV_US_. One could speculate that muscle shape changes slightly in different positions and that US-derived MT measured in the same position as the reference method would make the predictions better, thereby increasing the validity of MV_US_.

Our eligibility criteria were narrow and therefore all the studies included had almost the same design. This can be regarded as a strength since it makes it easier to comprehend the results. Unfortunately, this is also a weakness since eleven out of twelve studies were conducted in the same country and many of those studies came from the same research group. This affected the strength of the evidence synthesis according to GRADE along with some descriptive data that were missing. Another limitation is that the number of studies including children in our study is small and just one of the included studies [32] examined a population with medical condition. Only one study [31] developed equations for healthy children which makes it difficult to draw any meaningful conclusion for prepubertal children or for populations with disorders affecting muscle volume and emphasizes the need for more studies regarding the validity of MV_US_ in this field.

The results from the present systematic review are interesting and applicable in both scientific and clinical settings, for example in the field of sports medicine where a change in muscle mass is often a main outcome. Franchi et al. [61] correlated MT with ACSA over a twelve-week period of resistance exercise and reported changes in vastus lateralis MT that significantly correlated with the changes in mid-thigh ACSA. Comparisons between changes in MV_US_ based on MT and MV_MRI_ in conjunction with a period of resistance exercise would be interesting for future studies.

Another topic for future research would be to study whether the location of the measuring site along individual muscles or muscle groups can improve the predictions. Today, MT is measured at one location for each segment. The location selected is intended to correspond to the point of maximal CSA of the muscle [25, 35]. Yamauchi et al. [62] did measurements of MT with MRI at 10% intervals of the individual quadriceps muscles and compared how well different MT locations predicted MV. They found site-specific variations for how well MT correlated with MV between individual quadriceps muscles. For example, at mid-length, correlations between femur length × MT and MV for individual muscles, ranged between r^2^ 0.73–0.96 [62]. Ogawa et al. [38] also compared different measuring sites along the medial anterior aspect of the thigh and found that, for the adductor muscle group, the more proximal sites were better correlated to MV. How the location of measuring sites, or the addition of extra measuring sites, can influence the validity of prediction equations based on MT is still an open question and an area for future research.

From a clinical point of view, the present study has listed all the segments, sex, and the derived equations and compiled them into Table 2, helping clinicians with a user-friendly reference card to estimate MV with the help of US. This may be particularly helpful when monitoring progress after injury or surgery and may assist in making return-to-play decisions by giving clinicians a quick and simple prediction of the athlete’s MV.

## Conclusions

We conclude that the validity of US-derived equations based on MT is specific to the populations from which it is developed. The agreement with MV_MRI_ is moderate with SEE ranging between 6 and 12% in healthy adult populations. Suggestions for future research are to investigate whether testing positions, the location of measuring sites or increasing the number of measuring sites could improve the validity of prediction equations.

## Data Availability

All data generated or analysed during this study are included in this published article.

## Abbreviations

ACL: Anterior cruciate ligament
ACSA: Axial anatomical cross-sectional area
BH: Body height
BW: Body weight
CINAHL: Cumulative index to nursing & allied health literature
CT: Computed tomography
DEXA: Dual-energy x-ray absorptiometry
GRADE: Group reading assessment and diagnostic evaluation
MeSH: Medical subject headings
ML: Muscle length
MRI: Magnetic resonance imaging
MT: Muscle thickness
MV: Muscle volume
MV_MRI_: The method for estimating MV measured with MRI based on ACSA
NCIT: Non-contractile intramuscular tissue
PCSA: Physiological cross-sectional area
PRISMA: Preferred reporting items for systematic reviews and meta-analyses
QUADAS: Quality assessment of diagnostic accuracy studies
SD: Standard deviation
SEE: Standard error of the estimate
SEM: Standard error of the mean
US: Ultrasonography
MV_US_: The method for estimating MV measured with US based on MT
